# Application of Deep-Learning Methods to Bird Detection Using Unmanned Aerial Vehicle Imagery

**DOI:** 10.3390/s19071651

**Published:** 2019-04-06

**Authors:** Suk-Ju Hong, Yunhyeok Han, Sang-Yeon Kim, Ah-Yeong Lee, Ghiseok Kim

**Affiliations:** 1 Department of Biosystems and Biomaterials Science and Engineering, Seoul National University, 1 Gwanak-ro, Gwanak-gu, Seoul 08826, Korea; hsj5596@snu.ac.kr (S.-J.H.); redstar316@snu.ac.kr (Y.H.); yskra@snu.ac.kr (S.-Y.K.); lay117@korea.kr (A.-Y.L.); 2 National Institute of Agricultural Sciences, Rural Development Administration, Jeollabuk-do 54875, Korea; 3 Research Institute of Agriculture and Life Sciences, Seoul National University, 1 Gwanak-ro, Gwanak-gu, Seoul 08826, Korea

**Keywords:** deep learning, convolutional neural networks, unmanned aerial vehicle, bird detection

## Abstract

Wild birds are monitored with the important objectives of identifying their habitats and estimating the size of their populations. Especially in the case of migratory bird, they are significantly recorded during specific periods of time to forecast any possible spread of animal disease such as avian influenza. This study led to the construction of deep-learning-based object-detection models with the aid of aerial photographs collected by an unmanned aerial vehicle (UAV). The dataset containing the aerial photographs includes diverse images of birds in various bird habitats and in the vicinity of lakes and on farmland. In addition, aerial images of bird decoys are captured to achieve various bird patterns and more accurate bird information. Bird detection models such as Faster Region-based Convolutional Neural Network (R-CNN), Region-based Fully Convolutional Network (R-FCN), Single Shot MultiBox Detector (SSD), Retinanet, and You Only Look Once (YOLO) were created and the performance of all models was estimated by comparing their computing speed and average precision. The test results show Faster R-CNN to be the most accurate and YOLO to be the fastest among the models. The combined results demonstrate that the use of deep-learning-based detection methods in combination with UAV aerial imagery is fairly suitable for bird detection in various environments.

## 1. Introduction

Monitoring wild animals to identify their habitats and populations is considered to be important for the conservation and management of ecosystems, as well as because human health can be significantly affected by these ecosystems. Moreover, in situations in which increasing numbers of wildlife species are at risk due to rapid habitat loss and environmental degradation, regular monitoring of wildlife is essential for the understanding of abnormal changes and for the management and conservation of the ecosystems [1]. Therefore, wildlife populations have been surveyed using several counting methods, such as the total ground count method, the line-transect count method, the dropping count method, and aerial count method. These methods are based on the use of human observation to directly count the birds in local areas, and then this information is used to estimate the size of the population in an entire area [2]. The total ground count, which counts all the targets in a given area, has the advantage of being a simple method, but it has the disadvantage of being labor-intensive because all the targets must be counted manually [3]. The line-transect count method, which estimates the total population by measuring the number and distance of targets [4,5], shows a small bias when experiments are well designed, but the confidence interval is large when applied to large area surveys of species with an uneven distribution [6]. The dropping count method which estimates the population by using the excrement left by the target species is known to be more accurate than the direct count methods, and it has been used to reflect long-term information. Therefore, the dropping count method has been widely used as a population count method for large animals such as African elephants [7,8,9]. However, this method is difficult to apply to small animals compared to large animals because of the size and properties of the excrement; thus it is necessary to consider the defecation rate according to the species and season [3].

The aerial count method also has been significantly considered since the 1920s because it allows precise counting in the target area, and it is possible to investigate areas that are difficult for humans to access. Therefore, researches on animal monitoring via aerial photography have been actively conducted [10,11,12,13,14,15,16,17]. However, aerial surveys using large manned aircraft are very costly, and funding problems arising from these high costs make long-term monitoring difficult [18]. In addition, aerial surveys are risky in that airplane accidents account for the highest percentage of job-related deaths among field biologists [19]. Therefore, several attempts to overcome these difficulties have led the studies on the automated aerial imaging system such as an unmanned aerial vehicles (UAVs) to reduce the cost of human input and working times. Recently, UAV is in the spotlight for various types of researches, especially in the applications of aerial photography. In particular, a lightweight UAV is considered to be more economical than a manned aircraft or large UAV because a lightweight UAV can be operated by fewer personnel with relatively lower proficiency and automated operation is possible. Aerial surveillance using a lightweight UAV is cost-effective, with little risk of injuries or death resulting from aircraft accidents. The advantages of lightweight UAVs have encouraged recent studies of wild animal detection with the aid of aerial photography [20,21,22,23].

Target count investigations in aerial photography have generally been performed using two methods: manual and automatic counting. In the case of manual counting during aerial surveys, sampling count methods such as transect, quadrat, and block are commonly used [24]. The aerial line-transect method, which estimates the population of the target by recording the perpendicular distance between target and flight path, is widely used as the aerial counting method [25]. The aerial surveys of various wild animals such as pronghorn [26], dolphin [27], deer [28], and bear [29] are performed using the aerial line-transect method. Despite its wide range of applications in the aerial surveys of various animals [30,31,32,33,34,35], the manual count is labor-intensive in respect to inspecting the aerial photographs. The automatic counting method drastically reduces the amount of labor and time required because a large number of images can be processed quickly by an image-processing algorithm and computing system as compared with the manual count method. Previous studies related to automatic bird detection using aerial photographs have mainly used representative image-processing methods. Gilmer et al. [10], Cunningham et al. [11], and Trathan [12] used spectral thresholding and filtering techniques, and Abd-Elrahman [20] developed a bird detection method using template matching. Liu et al. [23] counted birds using unsupervised classification and filtering methods. However, despite these studies being successful in terms of bird counting, most of them focused on limited images of specific species located in a particular environment. In addition, the number of images used in these studies was relatively small. Therefore, these methods show some limitations in terms of their ability to detect birds distributed across various environments.

More recently, researches of object detection have rapidly developed following the introduction of deep-learning-based methods into object-detection applications. While existing machine-learning techniques require a feature selection process, deep-learning-based methods can learn features from given data by themselves. In addition, they perform well because of their deep-layer learning process that uses a large amount of data [36]. Among deep-learning methods, convolutional neural networks (CNN) are the most commonly used in deep-learning-based object-detection methods because they were optimally developed as classification networks suitable for image-type data [37]. CNN-based object-detection methods such as Region-based Convolutional Neural Network (R-CNN) [38], Fast R-CNN [39], and Faster R-CNN [40] consist of two stages: bounding box proposal and classification, which are processed sequentially. One-stage object-detection methods, such as You Only Look Once (YOLO) [41], Single Shot MultiBox Detector (SSD) [42], and Retinanet [43], also process the bounding box and classification processes simultaneously. One-stage processing methods are commonly known to be faster than two-stage methods; however, the performance in terms of the computing speed and accuracy for these two methods is different because the performance also depends on the type of CNN architecture the methods employ, e.g., Alexnet [40], Googlenet (Inception) [44], VGGNet [45], Squeezenet [46], Resnet [47], or Densenet [48].

The performance of deep-learning-based object-detection methods has been demonstrated to be higher than that of machine-learning-based methods, and the former of these two methods has rapidly improved in recent years. Therefore, deep learning has been actively applied to the research of sensing applications. Ammour et al. [36] conducted a car detection study using aerial photographs with CNN, and a support vector machine (SVM). Chang et al. [49] studied pedestrian detection from aerial photographs by developing the YOLO v.2 model, and Chen et al. [50] evaluated the Faster R-CNN method to detect airports from aerial photography. In addition, several studies are being conducted to investigate the use of deep-learning applications for wildlife monitoring with the aid of aerial photographs. Maire et al. [51] showed the feasibility of using the simple linear iterative clustering (SLIC) and CNN methods for the detection of wild marine mammals, and Guirado et al. [52] detected whales using satellite images in combination with a CNN-based method to detect the presence of whales and for whale counting.

Many interests and research efforts have been devoted to monitor wild birds for various purposes such as habitat and population investigation, and ecosystem conservation. In this study, deep-learning-based bird detection models are created and estimated using UAV aerial photographs. We therefore constructed a dataset containing aerial photographs of wild birds and bird decoys in various environments including lakes, beaches, reservoirs, and farms in South Korea, and employed five different deep-learning-based object-detection methods to analyze the UAV aerial photographs. Moreover, the performance of the proposed bird detection models was verified by comparing their computing speed and average precision (AP).

## 2. Materials and Methods

**Figure 1:**
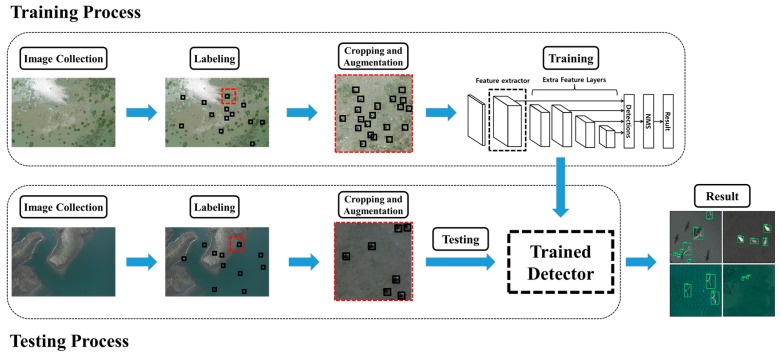
Flowchart of the proposed deep-learning method for bird detection.

Figure 1 shows the proposed method for bird detection, which consists of four stages. In the first stage of study, we capture the aerial photographs of both the wild birds and bird decoys using a UAV. This is followed by a labelling process by determining the pixel size of one box, which corresponds to one bird as 40 × 40 pixels. In the third stage, image preprocessing such as image cropping and augmentation are employed to obtain several hundred sub-images of the aerial photographs. The fourth step is devoted to the training process via feature representation learned by the hidden layers of each deep-learning model. Finally, the bird detection performance of each learning model is evaluated through a testing process.

### 2.1. Compilation of Aerial Photograph Dataset

**Figure 2:**
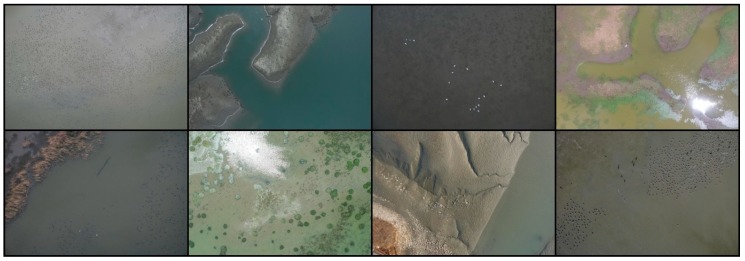
Aerial photographs of wild birds taken at an altitude of 100 m.

**Figure 3:**
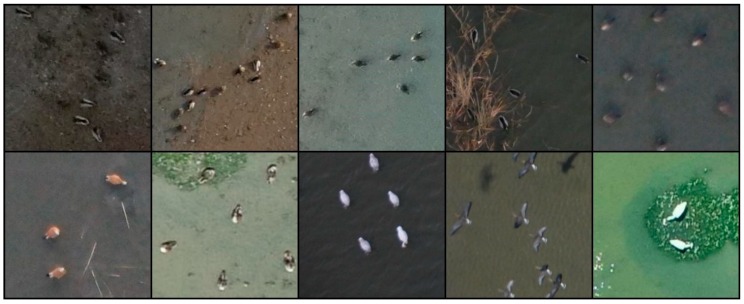
Enlarged images of wild birds taken at an altitude of 100 m.

Aerial photographs of wild birds were taken at Shihwa Lake (Incheon, Republic of Korea) and Yeongjong Island (Siheung, Republic of Korea) using a commercial UAV (K-mapper, SISTECH Inc., Seoul, Korea), as both of these locations are well-known wild bird habitats. The dimensions and maximum takeoff loads of the UAV were 750 mm × 750 mm × 250 mm and 4.5 kg, respectively. In addition, a color camera (NX-500, Samsung Corp., Republic of Korea) with a resolution of 6480 × 4320 pixels was attached to the UAV to capture aerial photographs at a flight altitude of 100 m. The camera specifications and shooting conditions are provided in Table 1. The aerial photographs usually included the spot-billed duck (*Anas poecilorhyncha*), green-winged teal (*Anas crecca*), great egrets (*Ardea alba*), and gray heron (*Ardea cinerea*). The collection process enabled us to obtain 393 aerial photographs from which images of 13,986 wild birds were prepared. Figure 2 shows representative aerial photographs of wild birds taken at an altitude of 100 m, and Figure 3 shows enlargements of those in Figure 2 to confirm the existence of wild birds.

**Figure 4:**
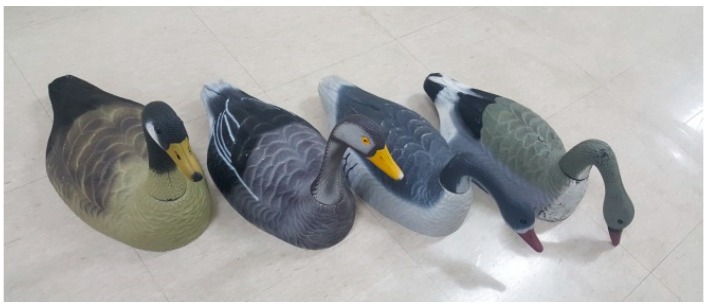
Four different kinds of bird decoys (ducks) used in aerial photography.

**Figure 5:**
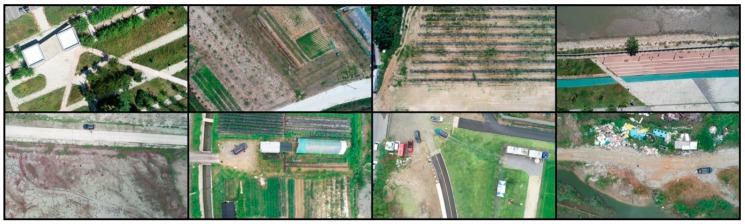
Aerial photographs of bird decoys taken at an altitude of 50 m.

**Figure 6:**
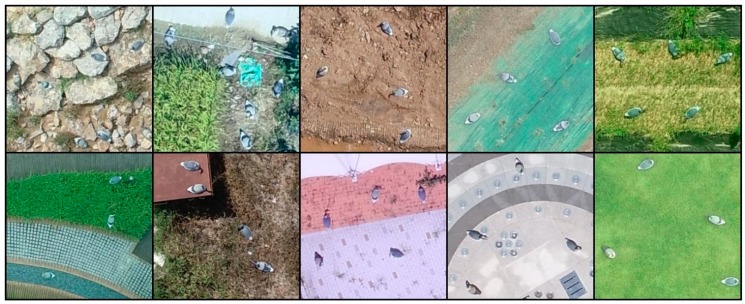
Enlarged images of bird decoys taken at an altitude of 50 m.

Additionally, aerial photographs of bird decoys were obtained in 15 different places, such as on farms, in parks, and in areas containing a reservoir, to enable a more robust learning process by adding images of bird decoys to the aerial photograph dataset. Four different kinds of bird decoys were used in the collection process, as shown in Figure 4. Aerial photographs of the bird decoys were acquired by another UAV (Phantom 4 Pro, DJI Co., Shenzhen, China), and a color camera with a resolution of 5472 × 3078 pixels was used for image acquisition. The altitude for the aerial photography of the bird decoys was adjusted by 50 m to synchronize the pixel size of a bird decoy to that of a wild bird. The specifications of the camera and the shooting conditions are also included in Table 1. In total, 169 aerial photographs, including 2,584 images of bird decoys were collected. Figure 5 shows representative aerial photographs of the bird decoys, and Figure 6 shows enlargements of those in Figure 5 taken in various environments.

### 2.2. Preprocessing and Augmentation of Dataset

Object detectability can be determined by the size of the target object in an image; therefore, the detection limit defined by the minimum size that can be detected is commonly regarded as an important performance indicator when several detection techniques are studied [53]. Especially in the case of the imaging process of aerial photographs, the size of the object of interest is generally much smaller than the size of the aerial photograph itself. Thus, image-preprocessing methods such as image cropping or increasing the network input size are necessary to enhance the detection performance of target objects from aerial photographs.

The datasets containing the aerial photographs used in this study consisted of images with two sizes, i.e., 6480 × 4320 pixels for the aerial photographs of wild birds and 5472 × 3078 pixels for the aerial photographs of bird decoys. The pixel size of one bird in each aerial photograph was calculated as approximately 40 × 40 pixels, and the size of these objects was such that it could rarely be detected without any image preprocessing. Therefore, we employed an image cropping method, and obtained 233 sub-images of wild birds and 139 sub-images of bird decoys for each aerial photograph. The size of each sub-image was adjusted as 600 × 600 pixels. In addition, it is generally known that the amount and diversity of data used for deep learning are very important to ensure that an object-detection model is robust; therefore, data augmentation methods are usually used as a preprocessing method in combination with deep learning [54]. We also employed an augmentation method to process aerial photographs to build a more robust model. The process of augmentation used in this study is as follows. First, we randomly selected a bird (object) in an aerial photograph, after which we produced a sub-image of 600 × 600 pixels with the selected bird included. If the center of another bird was included in this sub-image, the bird was considered to be included in the sub-image. Again, we randomly selected another bird that was not included in the previous sub-images, and then again produced a sub-image in the same way as before. This process was repeated until all the birds in the aerial photographs were included in sub-images. In the second step, all the sub-images were flipped vertically and horizontally with a probability of 50%, and both the Red, Green, Blue (RGB) value and contrast were also randomly re-adjusted with a probability of 50%. As a result of the image cropping and augmentation processes, we obtained 25,864 sub-images, including images of 137,486 birds, from the aerial photographs of wild birds, and 3143 sub-images, including images of 18,348 birds, from the aerial photographs of bird decoys, respectively.

### 2.3. Deep-Learning-Based Detection Methods

Lately, several deep-learning-based detection models have been developed and one of them is usually selected for application by considering the tradeoff between speed and accuracy [55]. Therefore, the optimal model and the approach followed to develop it should be carefully considered according to its applications. In this study, we employed five different deep-learning-based detection methods (Faster R-CNN, R-FCN, SSD, Retinanet, and YOLO), and evaluated the efficiency of the models by comparing their speed and accuracy.

Generally, R-CNN-based detection models consist of two stages, which are region proposal and region classification. Among the R-CNN-based models, the Faster R-CNN model is known to significantly reduce the speed while maintaining its performance by using a region proposal network (RPN) unlike R-CNN [38] and Fast R-CNN [39], both of which perform region proposal via a selective search process. Although several detection models have been proposed and developed to date, Faster R-CNN still performs excellently as regards accuracy, as confirmed in previous studies. The performance of Faster R-CNN, especially in the detection of small objects, is known to be superior to that of one-stage detectors. In addition, the R-FCN method is known to solve the translation-variance problem by introducing a position-sensitive score map; therefore, the computing speed of R-FCN is faster than that of Faster R-CNN, yet it maintains an accuracy similar to that of Faster R-CNN [40].

YOLO is a one-stage detection method in which localization and classification occur simultaneously in its network. It is designed to calculate the class probability for the grids of an image, the bounding boxes, and the confidence scores of each grid at the same time [41]. The performance of the YOLO model continues to improve and new versions such as YOLO v2 [56], which is based on Darknet-19 architecture, and YOLO v3 [57], based on Darknet-53 architecture, have been released, and they remain among the fastest one-stage detection methods. The SSD method was developed as a one-stage detection model that detects objects using a multi-scale feature map and a 3 × 3 × p small kernel [42], and this one-stage detection method, which is similar to YOLO, is well known to be very fast and highly accurate. Retinanet is another frequently used one-stage object-detection method that improves performance by enhancing the learning contribution to hard examples by introducing the concept of feature pyramid networks (FPN) and focal loss [43]. Even though it is a one-stage method, it occasionally outperforms existing two-stage-based detection models for its applications.

This study employed each of the aforementioned methods and their models were trained and tested by dividing the prepared images into three datasets: training, validation, and testing. The training dataset was used during the learning process of each model, and the validation dataset was used for fine-tuning to determine the optimal parameters of the model. The performance of each model was evaluated using the test dataset. In the case of previous studies, augmented samples were used only for training, but in our study, augmented images were also used for validation and testing to enhance the diversity and generality of the imaging environment during evaluation. In this study, the training dataset contains 19,366 and 2548 aerial photographs of wild birds and bird decoys, respectively, and these aerial photographs include images of 98,634 wild birds and 14,832 bird decoys, respectively. In total, 21,914 aerial photographs containing images of 113,466 birds were used for training. In the case of validation, the dataset contains 3412 and 435 aerial photographs of wild birds and bird decoys, respectively, and these aerial photographs include images of 17,842 wild birds and 2336 bird decoys. A total of 3847 aerial photographs and 20,178 bird images were used for validation. The test dataset has 3086 and 427 aerial photographs of wild birds and bird decoys, respectively, and it includes images of 15,378 wild birds and 2084 bird decoys. A total of 3513 aerial photographs and images of 17,462 birds were used for the test. Training and evaluation were carried out using a multi GPU embedded computing system (GeForce GTX 1080ti, Nvidia Corp., Santa Clara, CA, USA), and the tensorflow library [58].

The detection performance of the learned models were evaluated using average precision (AP), which is the most commonly used performance index for evaluating detection accuracy. AP is calculated as the area under the precision-recall graph with a predetermined detection threshold as described in Equations (1) and (2) (where *TP* is the true positive, i.e., the number of birds correctly detected, and *FP* is the false positive, i.e., the number of incorrect detections, and *FN* is the false negative, i.e., the number of ground truth birds undetected).

(1) $$\mathrm{Precision}=\frac{TP}{TP+FP}$$

(2) $$\mathrm{Recall}=\frac{TP}{TP+FN}$$

## 3. Results and Discussion

### 3.1. Test Results

**Figure 7:**
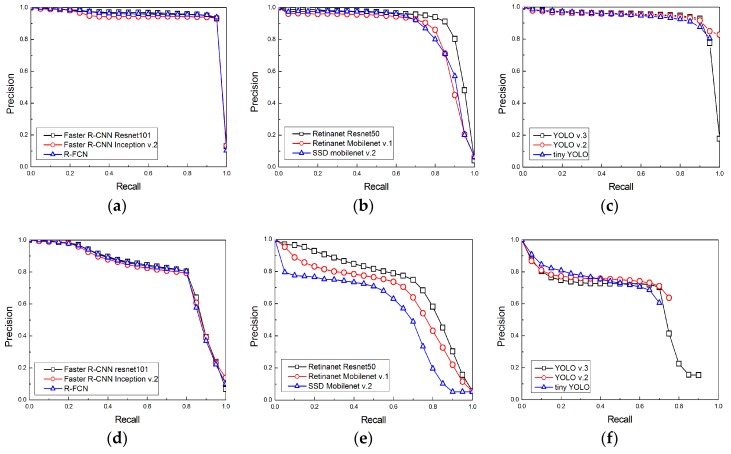
Precision-recall graphs of trained models: (**a**) Faster R-CNN-based models and R-FCN model for the intersection of union (IOU) threshold of 0.3; (**b**) SSD-based models for the IOU threshold of 0.3; (**c**) YOLO-based models for the IOU threshold of 0.3; (**d**) Faster R-CNN-based models and R-FCN model for the IOU threshold of 0.5; (**e**) SSD-based models for the IOU threshold of 0.5; (**f**) YOLO-based models for the IOU threshold of 0.5.

The intersection of union (IOU), defined as the ratio between the union and intersection of the detected box and the ground truth box, is used as an indicator to determine whether an object is correctly detected, and the average precision (AP) value changes according to the IOU threshold. In general, the AP is determined with an IOU threshold of 0.5, or by changing the IOU threshold from 0.5 to 0.95. In this study, it was occasionally observed that the ground truth boxes were inaccurately labeled as truth boxes that were very small compared to the entire aerial photograph, and this situation resulted in the IOU threshold being below 0.5 even though the bird was properly detected. Therefore, the AP of each model was evaluated by setting the IOU thresholds as 0.3 and 0.5. Figure 7 shows the precision-recall graph of each detection model.

**Figure 8:**
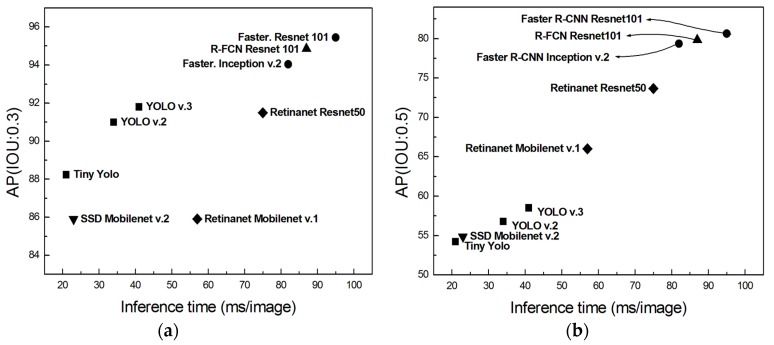
Performance comparison between average precision and inference time for all models: (**a**) IOU threshold of 0.3; (**b**) IOU threshold of 0.5.

Table 2 and Figure 8 present the test results for each model. In the case of the Faster R-CNN Resnet 101 model, the inference time was the slowest, 95 ms, but the best AP values of 95.44% and 80.63% were obtained for IOU thresholds of 0.3 and 0.5, respectively. Meanwhile, both the Faster R-CNN Inception v.2 model and R-FCN Resnet 101 model performed similarly for both speeds and AP values. In the case of SSD-based models, such as Retinanet Resnet50, Retinanet Mobilenet v.1, and SSD Mobilenet v.2, the AP values were comparatively high when the IOU threshold was 0.5, whereas the AP values were estimated to be lower than those of the one-stage detection models for the IOU threshold of 0.3. However, the performance of the YOLO models was relatively low for the IOU threshold of 0.5, whereas the AP value was estimated to be approximately 90% for the IOU threshold of 0.3, which was higher than that of SSD-based models. On the basis of the above results, it is observed that the SSD models and YOLO models show reverse performance for different IOU thresholds. This difference might be caused by the relatively lower object-detection performance of the SSD models despite their ability to label the ground truth boxes with good precision. Therefore, SSD models would need further adjustments to improve their performance.

**Figure 9:**
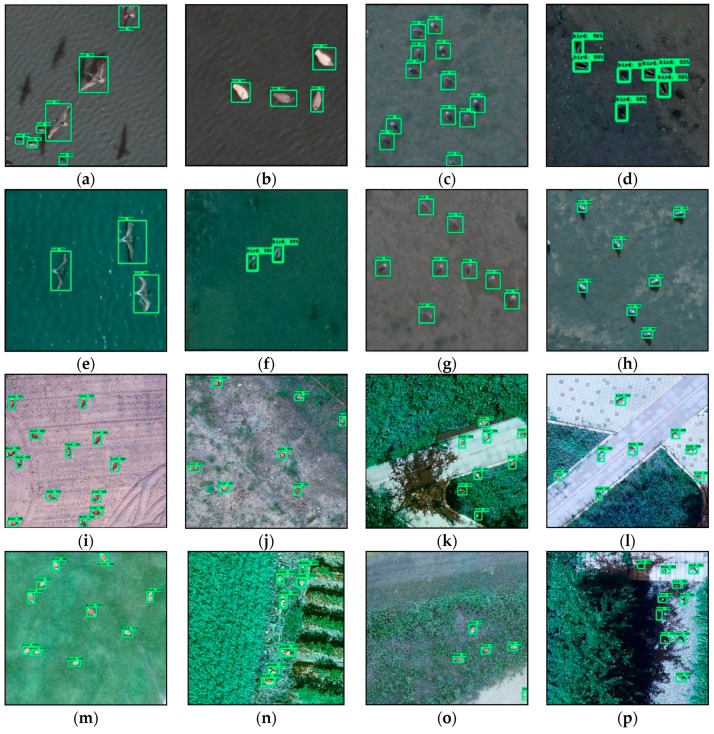
Bird detection results of Faster R-CNN Resnet 101 model. In (**a**,**e**), all birds in flight are successfully detected, whereas the black shadows of the flying birds remained undetected with a high accuracy, as shown as in (**a**). In addition, all birds in (**a**–**h**) are successfully detected regardless of the flying altitude of the birds.

Figure 9 shows the representative bird detection results of the Faster R-CNN Resnet 101 model, which shows the highest AP value among all the models in this study. Eight images (Figure 9a–h) show the detection results for wild birds, and those for the bird decoys are seen in Figure 9i–p. In Figure 9a,e, all birds in flight are successfully detected, whereas the black shadows of the flying birds remained undetected with a high accuracy, as shown as in Figure 9a. In addition, all birds in Figure 9a–h are successfully detected regardless of the flying altitude of the birds. In the case of the bird decoys, the birds are also accurately detected, as shown in Figure 9i–p, even though these birds are not easily recognizable to the human eye in Figure 9n,p. Especially in Figure 9p, it is demonstrated that the model can detect birds regardless of an illumination conditions.

**Figure 10:**
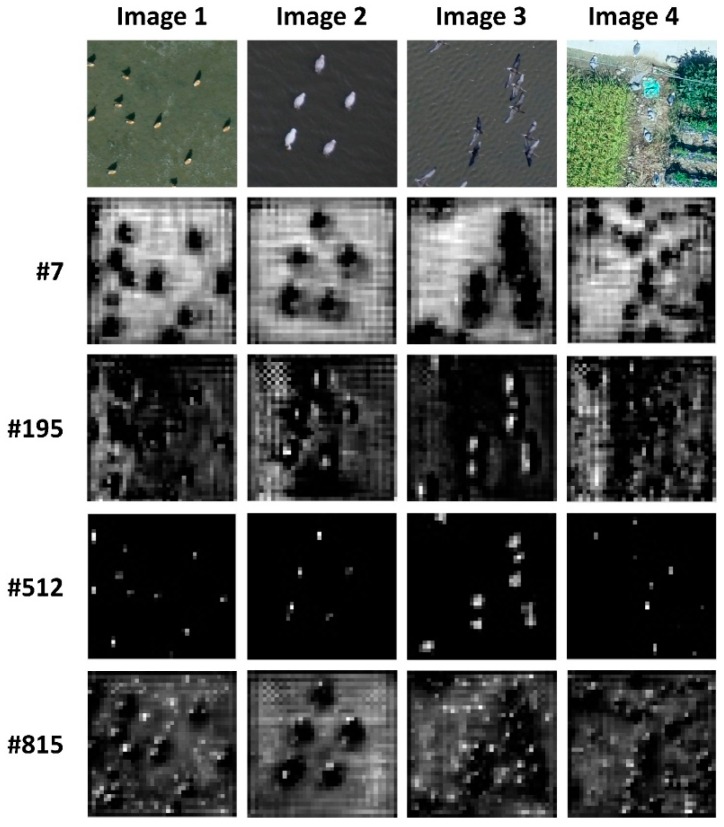
Visualization of representative feature map for the Faster R-CNN Resnet 101 model.

Figure 10 shows the representative visualization of the feature map derived from the first-stage feature detector of the Faster R-CNN Resnet 101 model. Each feature map from #7 to #815 includes different information, but it is observed that the area of the object appears in each feature map. Especially in feature map # 512, only the areas in which a bird exists is more clearly activated. In addition, in the case of Image 4, which has a more complicated background than others, the background is largely removed from the feature map during the feature representation learned by the hidden layers.

**Figure 11:**
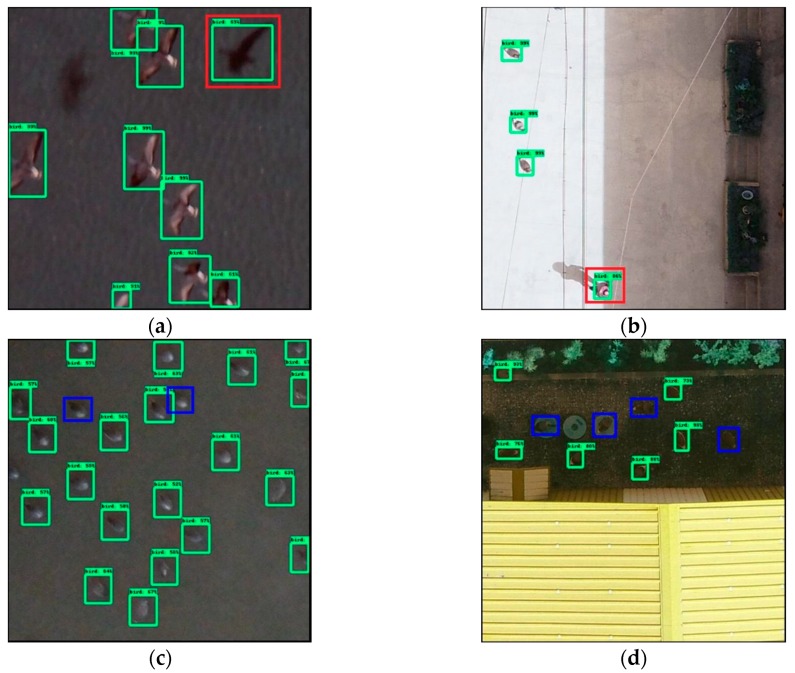
Erroneous detection results: (**a**) shadow of a bird detected as a bird; (**b**) a person wearing a straw hat detected as a bird; (**c**) no detected wild birds; (**d**) no detected bird decoys.

Figure 11 shows erroneous examples such as incorrect detection and no detection. Figure 11a,c are the erroneous results of wild birds, and those of bird decoys are presented in Figure 11b,d. In Figure 11a, an incorrect detection marked with the red box is detected as a flying bird but instead it is a shadow of a flying bird (false positives), and Figure 11b shows that the person wearing a straw hat is detected as a bird. Moreover, the blue boxes in Figure 11c,d indicate birds that are not detected by the detection model (false negatives). On the basis of these results, we assume that bird shadows, people, and rocks can cause erroneous detection (false positives), in addition, birds remained undetected if the background color is similar to that of the bird, or when the birds are obscured by a dark shadow (false negatives).

**Figure 12:**
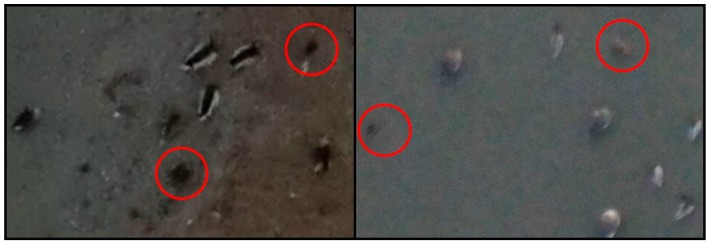
Aerial photographs including difficult objects to identify even with the human eye.

Figure 12 shows some cases whereby it is fairly difficult to distinguish whether they are birds or not, even with the human eye. Unfortunately, these cases can be the cause of error during the training and testing processes; therefore, the pixel size of a bird image needs to be larger to improve the detection performance. Moreover, lowering the flight altitude of the UAV for enhanced image resolution would increase the likelihood of the birds on the ground taking flight in response to the UAV. Therefore, using a camera with the best performance (an optimal view angle) is the best alternative to prevent this situation if the cost permits.

### 3.2. Counting Errors

In this study, the counting accuracy was calculated to evaluate the bird-counting performance for the aerial photographs of wild birds during a testing. For the models that obtained the highest AP values, such as the Faster R-CNN Resnet 101 model, Faster R-CNN Inception v.2 model, and R-FCN Resnet 101 model, the counting errors were analyzed to estimate their bird-counting performance. The counting accuracy, expressed by Equations (3) and (4), was considered as the sample mean error, which was analyzed as the average of the counting error for the test dataset (where $E_{i}$ is the counting error of the $i_{th}$ image, and $C_{i}$ is the actual number of birds in the $i_{th}$ image, and ${\hat{C}}_{i}$ is the number of birds in the $i_{th}$ image counted by the detection model, and *N* is the number of images) [1].

(3) $$\mathrm{sample}\;\mathrm{mean}\;\mathrm{error}\;=\;\frac{\sum_{i=1}^{N}E_{i}}{N}$$

(4) $$E_{i}=\left|\frac{C_{i}-{\hat{C}}_{i}}{C_{i}}\right|$$

From the results of counting accuracy, it is estimated that the Faster R-CNN Resnet 101 model has the best sample mean error of 4.6% which is higher than that of the Faster R-CNN Inception v.2 model and R-FCN Resnet 101 model by 1.1% and 0.8%, respectively. Previous studies [10,11,13,16,20] also showed a low sample mean error in the range 3.5%–10.5%; however, most of these studies focused on colonies of specific species, and the development and evaluation of detection methods were performed with small quantities of samples. Therefore, we expect the deep-learning-based detection models employed in this study to be potentially useful for the detection of wild animals using UAV aerial photography, especially in bird detection. In addition, our models also compliment the limitations of conventional image-processing methods.

## 4. Conclusions

This study employs five different deep-learning-based object-detection methods, i.e., Faster R-CNN, R-FCN, SSD, Retinanet, and YOLO, to create bird detection models using aerial photographs captured by UAV. On the basis of the experimental test results, the performance of the training models with respect to detecting birds is fairly good, with the AP values ranging from 85.01% to 95.44% (IOU thresholds: 0.3) during the test process. These results confirm that the proposed deep-learning-based model and its process is suitable for bird detection using UAV aerial photographs, and could ultimately be used for monitoring wild animals.

We also expect the deep-learning models developed in this study to be effective for other aerial photographs with a different field of view, resolution, sensor quality, flight altitude, etc. However, they would be restrictive because the pixel size of labelling box for one bird is predetermined to be approximately 40 × 40 pixels in this study. The use of the deep-learning models developed in this study for bird detection applications together with different aerial photography would require the pixel size of one bird in an aerial photograph to exceed 40 × 40 pixels to ensure successful detection. However, detection failure may increase if the pixel size of one bird obtained with different aerial photography is smaller than 40 × 40 pixels.

It is commonly known that aerial photographs are stitched together into one large aerial photograph with a predetermined percentage of image overlap. This stitching work is generally known as mapping; therefore, one bird could be present twice or more in a large aerial photograph, especially when birds are flying during UAV aerial photography. In our study, the mapping process is not included because our deep-learning models are devoted to working on individual aerial photographs during the whole process. Therefore, the deep-learning models built in this study may detect the same bird twice or more if the deep-learning models are used with other large aerial photograph in which the bird exists twice or more. This is because the purpose of the deep-learning models built in this study is to detect rather than count birds from aerial photographs. We suspect that the counting errors of deep-learning models is due to this.

Despite the above-mentioned limitations of our application, we expect it to be possible to adjust the deep-learning-based object-detection methods for use in various applications including bird counting, monitoring of migratory birds, and identification of wild bird habitats. Toward this goal, the optimization of deep-learning models should be continuously researched and developed for their specific applications with careful consideration of the speed–accuracy tradeoff. Our next study, which aims to develop a more robust detection model, is currently underway and involves collecting more diverse aerial photographs of birds including dead birds. In addition, as our next task, we plan to enhance the speed and accuracy by modifying the network structure of our models.

## Figures and Tables

**Table 1:** Camera specifications and shooting conditions used in the aerial photography.

	Wild Birds Imaging	Bird Decoys Imaging
Imaging camera	NX-500, Samsung corp.	Da Jiang Innovation (DJI) camera
Resolution	6480 × 4320 pixels	5472 × 3078 pixels
Focal length	35 mm	8.8 mm
Sensor size	23.5 × 15.7 mm	13.2 × 8.8 mm
Altitude	100 m	50 m
Field of View (FOV)	67.1 m × 44.9 m	81 m × 45.6 m
Ground Sample Distance (GSD)	0.0104 m/pixel	0.0148 m/pixel

**Table 2:** Test results of detection models.

Meta Architecture	Feature Extractor	Inference Time (ms/photograph)	AP		AP_wild_	AP_model_
			IOU:0.3	IOU:0.5	IOU:0.3	IOU:0.3
Faster R-CNN	Resnet 101	95	95.44	80.63	96.18	95.23
	Inception v.2	82	94.04	79.35	95.90	93.94
R-FCN	Resnet 101	87	94.86	79.83	95.92	94.12
Retinanet	Resnet 50	75	91.49	73.66	92.37	83.75
	Mobilenet v.1	57	85.01	66.01	90.05	62.64
SSD	Mobilenet v.2	23	85.90	54.87	89.13	65.20
Yolo v3	Darknet-53	41	91.80	58.53	91.98	90.77
Yolo v2	Darknet-19	34	90.99	56.80	92.34	88.99
	Tiny Yolo	21	88.23	54.22	89.75	79.24
